# HPTLC and ATR/FTIR Characterization of Antioxidants in Different Rosemary Extracts

**DOI:** 10.3390/molecules26196064

**Published:** 2021-10-07

**Authors:** Snezana Agatonovic-Kustrin, Ksenia S. Balyklova, Vladimir Gegechkori, David W. Morton

**Affiliations:** 1 Department of Pharmaceutical and Toxicological Chemistry named after Arzamastsev of the Institute of Pharmacy, I.M. Sechenov First Moscow State Medical University (Sechenov University), 119991 Moscow, Russia; balyklova_k_s@staff.sechenov.ru (K.S.B.); vgegechkori@gmail.com (V.G.); d.morton@latrobe.edu.au (D.W.M.); 2 School of Pharmacy and Biomedical Sciences, La Trobe Institute for Molecular Sciences, La Trobe University, Edwards Rd, Bendigo 3550, Australia

**Keywords:** rosemary antioxidants, HPTLC fingerprints, fermentation assisted extraction, fractional freezing, abietic acid, natural eutectic solvent

## Abstract

The effect of spontaneous fermentation by lactic acid bacteria on the extraction yield of bioactive compounds and antioxidant activity from rosemary leaf extracts was investigated using high-performance thin-layer chromatography (HPTLC). Brining and spontaneous fermentation with lactic acid bacteria more than doubled extraction of polyphenolics and antioxidants from the rosemary leaves. The results show that lactic acid fermentation enhances antioxidant activity in extracts by increasing the total phenolic content but does not increase extraction of phytosterols. Increased extraction of phenolic oxidants during fermentation assisted extraction, results from the in situ generated natural eutectic solvent from the plant sample. ATR-FTIR spectra from the bioactive bands suggests that this increased antioxidant activity is associated with increased extraction of rosmarinic acid, depolymerised lignin, abietane diterpenoids and 15-hydroxy-7-oxodehydroabietic acid.

## 1. Introduction

Plant phenolics, the most abundant secondary metabolites, are the main source of dietary antioxidants and have many health benefits [1]. Rosemary and other members from the Lamiaceae family are a rich source of natural antioxidants. The European Union has recently approved rosemary extracts as a safe and effective natural antioxidant for food preservation [2]. However, different extraction methods were found to recover quite different groups of polyphenols. Furthermore, their use in foods is limited because of their odour, colour and taste. For that reason, various methods have been developed for the preparation of odourless and colourless antioxidant compounds from rosemary. The principal antioxidant components in these extracts are phenolic diterpenes carnosol and carnosic acid, and phenolic acids such as rosmarinic acid (the caffeoyl derivative) [3,4]. Rosmarinic acid is used as an antioxidant agent in foods and as a scent in cosmetics [5]. The solubility of phenolics depends on their chemical nature and the polarity of the extraction solvent. Rosemary extracts are usually prepared from dried rosemary leaves with 2% to 26% yield depending on the solvent and extraction method [6]. Soxhlet extraction was found to be the most efficient method, followed by dry leaf decoction [7]. An increase in the extraction time and temperature increases the solubility of the phenolics, as decreased solvent viscosity and the surface tension helps the solvents to reach the plant cell matrix and improve the extraction rate. However, long extraction times and high temperature might increase oxidative degradation resulting in a decrease in the yield of phenolics in the extracts [8].

The aim of this study was to investigate the effect of spontaneous fermentation by lactic acid bacteria on the extraction yield of bioactive compounds and antioxidant activity from rosemary leaf (*Rosmarinus officinalis L.,* Lamiaceae) extracts, using high-performance thin-layer chromatography (HPTLC) and attenuated total reflectance-Fourier transform infrared (ATR-FTIR) spectroscopy to provide chemical characterisation of detected major antioxidants. It is known that the fermentation process induces a structural breakdown of plant cell walls, leading to the release of many antioxidants [9,10]. Since measuring the total phenolic content can be incorrect as the compositions of specific phenolic components in the rosemary extracts are not identified, chromatographic separation of the phenolic and diterpenoid antioxidants was performed.

HPTLC is an established method for the analysis of medicinal plants and the only chromatographic method that can present the results as colourful images. Furthermore, HPTLC enables parallel instead of sequential analysis, and thus can document multiple fingerprints for comparison on the same plate image. Fingerprints can be visualized under white light, under UV 366 nm and UV 254 nm, and can be enhanced by derivatization processes. In comparison, the HPLC method, although being the most common chromatographic method used for quantitative analysis of rosemary extracts, requires either longer analysis times, leading to degradation of unstable phenolics, or incomplete baseline separation, which makes quantitation of the phenolic compounds in the extract difficult [11].

Although mass spectrometry (MS) hyphenated with HPTLC, is usually used to identify separated components [12], FTIR spectroscopy provides high sensitivity, information-rich mid-IR spectra with no sample preparation [13]. The ATR sampling method enables rapid chemical characterisation of compounds within a short timeframe.

## 2. Results and Discussion

The effect of spontaneous lacto-fermentation on the extraction efficiency of antioxidants and natural products from rosemary leaves was studied via HPTLC profile comparison. The pH of the fermentation broths declined from pH 7.4 to 3.8–3.5 after three days of incubation, indicating the formation of lactic acid, and remained constant over the entire fermentation period [14]. Low pH prevents other pathogenic bacteria to grow, while different cell wall degrading enzymes from lactic acid bacteria (LAB) helped in the maceration of the plant matrix and promoted the release of the plant secondary metabolites. This biotransformation of complex constituents into extractable components during fermentation is similar to the metabolic activity of the gut microbiota in the human intestine, with its microbiome being composed of a wide range of different LAB [15]. Increased extraction of polyphenolic oxidants during fermentation assisted extraction from rosemary leaves, may also result from the in situ generated eutectic solvent from the plant sample [16]. Lactic acid produced and released during fermentation may combine with amino acids naturally present in rosemary leaves and stems [17] to form a natural deep eutectic solvent (NADES). Lactic acid-based natural deep eutectic solvents (NADES) are very efficient in extraction of polyphenolic antioxidants from medicinal plants [18].

The ethyl acetate extract from nonfermented dried leaves was compared with ethyl acetate extracts from fermented dried and fermented fresh rosemary leaves, in terms of antioxidant activity, total phenolic content, and extraction of natural products, via HPTLC screening analysis. Ethyl acetate was selected as the most efficient solvent for extraction of the widest range of polyphenolics and phytosterols [19]. It is a moderately polar solvent that has the advantages of being volatile, relatively non-toxic, and non-hygroscopic. The percentage yield of extracts was 5.7% (1.02 g/17.85 g) for unfermented extract from dried leaves, 5.27% (1.58 g/30 g) for fermented extract from fresh leaves, and 3.53% (1.06 g/30 g) for fermented extracts from dried leaves.

**Figure 1:**
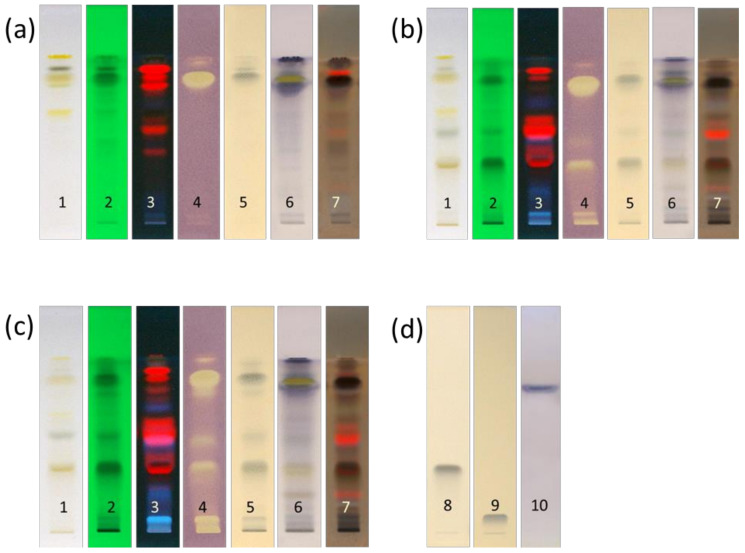
HPTLC fingerprints of (**a**) unfermented extract, (**b**) fermented extract from fresh leaves, (**c**) fermented extract from dried leaves, and (**d**) standards. Track 1, white light; track 2, UV 254 nm; track 3, UV 366 nm; track 4, DPPH· assay; track 5, with ferric chloride; track 6, with anisaldehyde/sulfuric acid under white light; track 7, with anisaldehyde/sulfuric acid under UV 366 nm; track 8, caffeic acid standard with ferric chloride; track 9, rosmarinic acid standard with ferric chloride; track 10, β-sitosterol standard with anisaldehyde/sulfuric acid.

**Figure 2:**
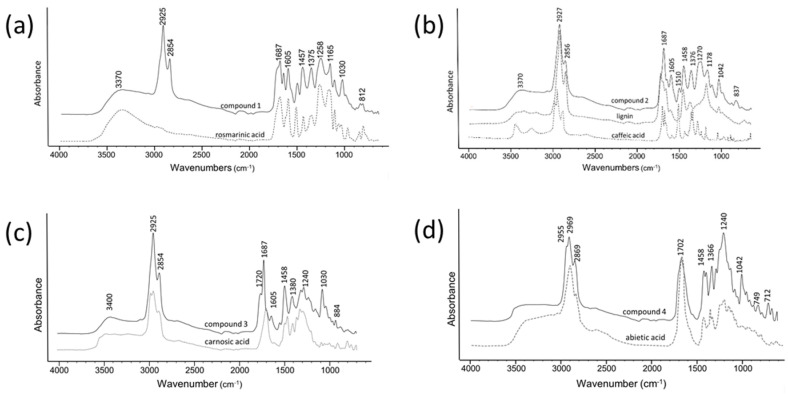
Superimposed ATR-FTIR spectra of: (**a**) compound from a band at *R*_F_ = 0.10 (black line) and rosmarinic acid; (**b**) compound from a band at *R*_F_ = 0.37 (black line), lignin and caffeic acid; (**c**) compound from a band at *R*_F_ = 0.53 (black line) and carnosic acid; (d) compound from a band at *R*_F_ = 0.83 (black line) and abietic acid.

Extracts were separated on HPTLC plates using a mobile phase optimised for separation of components. It consisted of *n*-hexane, ethyl acetate, and acetic acid (60:36:4 *v/v/v*). After component separation, images of the plates were taken both before and after plate derivatization. A rapid TLC-DPPH· antioxidant assay was used to screen for free radical scavengers, i.e., components in rosemary extracts that can donate an electron and scavenge the stable DPPH free radical. Derivatization with ferric chloride was used to detect compounds with phenolic groups (Figure 1), while derivatizations with anisaldehyde/sulfuric acid reagent was used to visualize natural products, especially terpenoids [20]. Rosemary is well known for its production of polyphenolic diterpenes which contribute to antioxidant activity of rosemary extracts, mostly carnosic acid (Figure 2b) and carnosol [21], and rosmarinic acid (a cinnamic acid derivative) [22].

Compounds with antioxidant activity were visualized as pale-yellow zones against a purple background in the DPPH· assay, while compounds with a phenolic group were seen as coloured bands, usually blue and violet, after derivatization with ferric chloride. Phenols form violet or blue coloured complexes with ferric chloride, while flavonoids form a green-blue colour [23]. Phenolic acids such as caffeic acid and rosmarinic acid produce grey-green spots, as seen in fermented samples at the lower part of the chromatograms. The blue band at *R*_F_ = 0.10 in fermented extracts has an identical migration distance to the rosmarinic acid standard, while the blue band at *R*_F_ = 0.37 has the same migration distance as the caffeic acid standard. The presence of rosmarinic acid was confirmed under UV light before derivatization, as this active zone showed a native blue fluorescence in the chromatogram illuminated at UV 366 nm. Cinnamic acid derivatives, such as rosmarinic and caffeic acids, give characteristic light blue fluorescence under UV 366 nm as seen in Figure 1b,c track 3 at *R*_F_ = 0.10 for rosmarinic acid, but this could not be confirmed at *R*_F_ = 0.37 for caffeic acid.

*p*-Anisaldehyde/sulfuric acid regent produces a range of colour differentiation, from grey blue, through violet to green with natural products. Blue bands are typical for monoterpenes and monoterpene alcohols, dark purple for triterpenes and phytosterols, grey for steroids and terpene esters [24,25], and brown spots for diterpenes [26]. After derivatization with *p*-anisaldehyde/sulfuric acid regent, purple and violet bands appeared in the upper part of the ester zone, while brownish and blue bands were seen only in fermented samples in the first half of the chromatograms (*R*_F_ = 0.30–0.50). The purple band at *R*_F_ = 0.76 has been assigned to the presence of phytosterols, due to the same colour and position in the chromatogram as the β-sitosterol standard.

Gallic acid, a strong triphenolic antioxidant, was used as a reference analyte to quantify antioxidants in the DPPH· assay and to quantify the total phenolics content in the ferric chloride assay. Antioxidant activity and total phenolic content were expressed in gallic acid equivalents (GAE). A sitosterol standard was used as a reference standard to quantify the total natural products extracted, with values expressed in sitosterol equivalents (SE). A rosmarinic acid standard curve was used as a reference to quantify antioxidants at *R*_F_ = 0.10 in chromatograms, and to establish how fermentation affects extraction of rosmarinic acids. This was expressed in rosmarinic acid equivalents (RAE).

**Figure 3:**
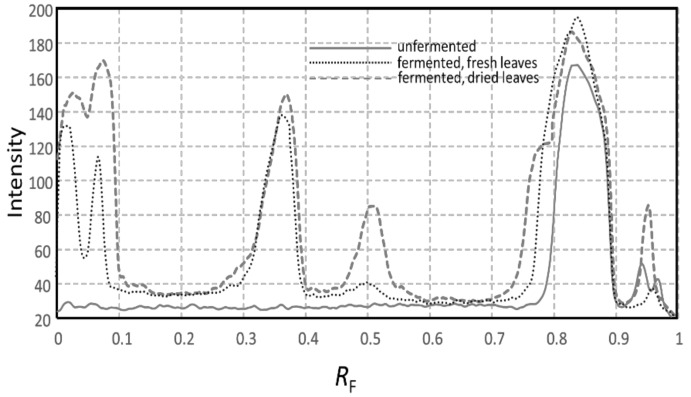
Comparisons of the DPPH· extract bioautograms.

It is known that fermentation helps to break down plant cell walls leading to the release of many antioxidants [9,10]. The antioxidant properties of extracts, seen as pale-yellow zones against purple background on the HPTLC plate in the DPPH· assay, were significantly increased after fermentation (Figure 3), from 2.92 µg of GAE in unfermented extract to 7.36 µg of GAE per 20 µL of the dried fermented leaf extract (Table 1). The results show that fermentation increases release and extraction of phenolic acids from the plant material as the total phenolic content increases from 29.22 to 75.57 µg of GAE (Table 1). As expected, the free radical scavenging activities of extracts in the DPPH· assay, were highly correlated to total phenolic content, with a correlation coefficient of *r* = 0.98.

The amount of phenolics at *R*_F_ = 0.10 in fermented extracts was quantified in rosmarinic acid equivalents, by comparing the areas of blue bands at *R*_F_ = 0.1 after derivatization with ferric chloride (Table 2), using a rosmarinic acid calibration curve (Table 3). The amount of phenolics at *R*_F_ = 0.37 in fermented extracts, was quantified in caffeic acid equivalents (CAEs) (Table 2), by using a caffeic acid standard curve (Table 3), and by comparison of the areas of dark bands under 254 nm against a green background. The amount of phytosterol present at *R*_F_ = 0.76 was quantified using sitosterol equivalents by comparing the areas of purple bands after derivatization with anisaldehyde/sulfuric acid reagent with the β-sitosterol calibration curve (Table 3).

Lactic acid bacteria fermentation has a significant effect on the antioxidant activity and phytochemical composition of the extracts. The content of antioxidants and free phenolics are almost doubled in fermented extracts from fresh leaves and more than doubled in fermented extracts from dried leaves. However, although fermentation has improved the antioxidant activity in extracts by increasing the total phenolic content, it did not significantly affect the extraction of other natural products in the extracts, especially phytosterols. The areas of the purple bands at *R*_F_ = 0.76, after derivatization with anisaldehyde/sulfuric acid, which correspond to the β-sitosterol standard, were not significantly changed by fermentation (Table 2).

Compounds responsible for the significant increase in antioxidant activities in fermented extracts were characterised by detailed analysis of the ATR-FTIR spectra from the bioactive zones at *R*_F_ = 0.10, 0.37, 0.53 and 0.83.

**Figure 4:**
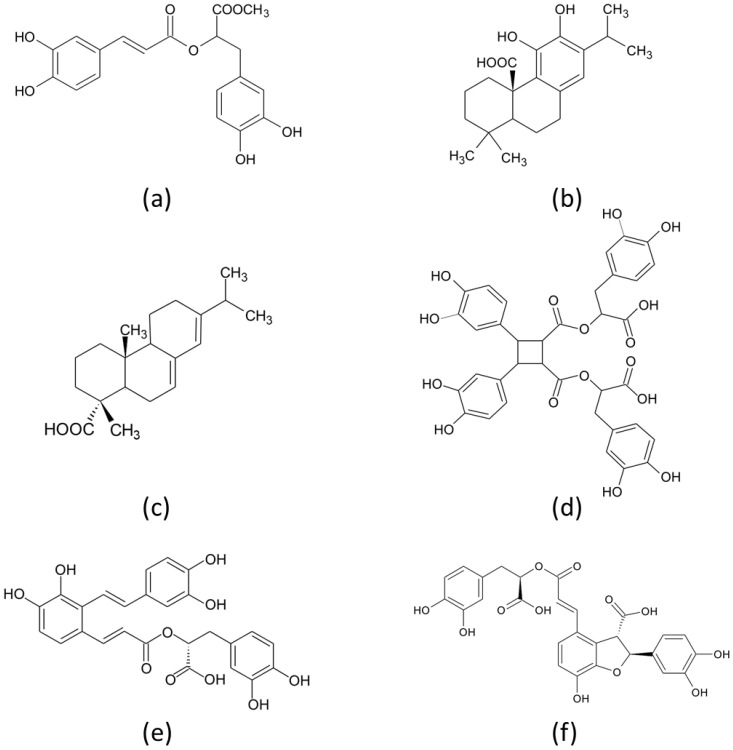
The chemical structures of: (**a**) rosmarinic acid methyl ester, (**b**) carnosic acid, (**c**) abietic acid, (**d**) sagerinic acid, (**e**) salvianolic acid A, and (**f**) lithospermic acids.

The compound isolated from the band with the same *R*_F_ = 0.1 (compound **1**) as rosmarinic acid (Figure 2a), also exhibits a similar ATR-FTIR spectrum to the rosmarinic acid standard (Figure 2a), suggesting it is a rosmarinic acid derivative. The broad bands observed at 3370 cm^−1^ are assigned to O-H stretching vibrations. The O-H in-plane-bending deformation vibration for phenol is in the region 1150–1250 cm^−1^ [27]. For the rosmarinic acid molecule, the OH in-plane bending vibration appears as a very strong band in at 1358 cm^−1^. A maximum for esters at 1725 cm^−1^ is missing and is seen only as a shoulder peak. Instead, there is a maximum at 1687 cm^−1^ for a C=O group [28,29]. The absorption peak at 1655 cm^−1^ is ascribed to the stretching vibration of the C=O in the conjugated carboxylic acid, and 1605 cm^−1^ is ascribed to the ring (C=C aromatic). The ring C-C stretching vibrations occur in the region 1625–1430 cm^−1^. Four bands of variable intensity were observed at 1605, 1509, 1457, and 1358 cm^−1^ [30]. Since rosmarinic acid has one methylene group in an adjacent position to the second ring, the C-H stretching vibrations of the methylene group are much lower than those of the aromatic C-H ring stretching (Figure 2a, dashed line). The strong bands at 2925 cm^−1^ and 2854 cm^−1^ are characteristics of alkyl chains, suggesting a methyl ester of rosmarinic acid (Figure 4a). Rosmarinic acid is the simplest caffeic acid dimer [31], an ester of caffeic acid and 3,4-dihydroxyphenyllactic acid. It has a lignin monolignol-like structure and is ultimately converted into salvianolic acid B, during phenolic acid metabolism in the plant [32]. Sagerinic acid (derived from rosmarinic acid), salvianolic acid A and B, and a pentacyclic triterpenoid corosolic acid were recently detected in rosemary for the first time [7]. Sagerinic acid is a rosmarinic acid dimer (caffeic acid tetramer), while salvianolic acid A and B and lithospermic acid A and B, are caffeic acid trimers [33].

However, the compound from the band with the same *R*_F_ = 0.37 (compound **2**) as caffeic acid, shows a similar ATR-FTIR spectrum to the spectrum of lignin (Figure 2b) [26,34]. Hydroxycinnamic acids are common in plants. They occur less often as the free acids, such as caffeic acid, but generally occur as either soluble conjugated compounds, such as chlorogenic acids, and less widely as derivatives such as rosmarinic acid in rosemary. They have antioxidant, antimicrobial, and antifungal properties, and also function as structural components of cell walls in woody plants [35], being precursors of lignans and lignin [36]. Thus, compound **2** could be a solubilized depolymerised lignin. Rosemary and thyme are known for their high amounts of caffeic acid [37], a key intermediate in the biosynthesis of lignin. Lignin is synthesized through oxidative polymerization/coupling of 4-hydrophenylpropanoids (the monolignols). Hydroxycinnamic derivatives, such as caffeic acids, are involved in the phenylpropanoid pathway and cell wall lignification [38]. Modification of monolignols through hydroxylation and O-methylation regulates the chemical and physical properties of the lignin polymer. For spectral comparison, lignin was extracted from rosemary leaves according to the CIMV (Compagnie Industrielle De La Matiere Vegetale) process which uses a formic/acetic acid mixture [39]. At present, the Kraft pulping process [40] is the most widely used method for lignin extraction and occurs under basic conditions, in contrast to the CIMV process which extracts the lignin under acidic conditions. The ATR-FTIR spectrum of sample 3 exhibits a broad band between 3500–3100 cm^−1^, which is assigned to phenolic hydroxyl group OH stretching vibrations. Bands at 2927 and 2856 cm^−1^ are assigned to C-H stretching vibrations of the methoxyl group, and the shoulder peak at 3034 cm^−1^ to the aromatic C-H stretch. The absorption bands at 1687 and 1655 cm^−1^ are attributed to the conjugated C=O band from the aromatic carboxyl groups [41,42]. The absence of absorption bands for stretching vibrations of C=C bands at 1626–1608 cm^−1^, and the low intensity of the band at 970 cm^−1^, indicates a small amount of C=C double bonds present. Absorption bands at 1605 cm^−1^ and 1510 cm^−1^ are related to vibrations of aromatic rings present in lignin [43]. The band at 1457 cm^−1^ can be assigned to both CH_2_-, and OCH_3_- groups. The absorption band at 1457 cm^−1^ is related to CH_3_ in acetyl groups. The absorption bands at 1375 cm^−1^ can be assigned to phenolic hydroxyls, and absorption bands at 1365 cm^−1^ to symmetric deformation vibrations of C-H in methoxy groups [44]. The intensity increase of the absorption band between 1270–1230 cm^−1^, as seen in the spectra of methylated and acetylated lignin, allows us to relate this band to asymmetric stretching vibrations of the C-O-C linkages in phenolic ethers (C-O stretch) and esters or to phenolic hydroxyls [44]. The bands at 1178, 1125 and 1042 cm^−1^ originate from methoxyl groups, while the absorption bands, located in the region 900–700 cm^−1^, are caused by deformation vibrations of C-H bonds in the benzene ring [45].

The similarity of the ATR-FTIR spectrum of the compound isolated from the band at *R*_F_ = 0.53 (compound **3**) and the spectrum of carnosic acid (Figure 4b), suggests the presence of an abietane type diterpene (Figure 2c). The C-H stretch from methylene groups at 2925 cm^−1^ (asymmetric stretching) and 2854 cm^−1^ (symmetric stretching) could be caused by the hydrocarbon skeleton of the diterpene three ring structure [46]. The bands at 1719 cm^−1^ and 1687 cm^−1^ can be attributed to the stretching of the C=O bond in carboxylic acids. The skeletal vibrations of the aromatic ring (C-C- stretches) are observed at 1605 cm^−1^, 1508 cm^−1^, 884 cm^−1^, 821 cm^−1^ and 750 cm^−1^. The bands at 1605 cm^−1^ and 1458 cm^−1^ are due to symmetric structure of ring [47]. Bending of the C-H bond out of the plane (oop) of the ring at 821 cm^−1^ and 750 cm^−1^ is weaker, due to the higher level of hydrogenation of diterpenic skeleton [48]. The bands at 1388 cm^−1^ and 1366 cm^−1^ refer to an isopropyl (-C(CH_3_)_2_) group due to CH_2_ and CH_3_ bending, while the C-O asymmetric stretching of COOH is observed at 1240 cm^−1^ and 1185 cm^−1^. An O-H deformation band appears at 1030 cm^−1^. Carnosic acid, an abietane-type diterpene is also a commercially available antioxidant. Carnosol and carnosic acid account for over 90% of the rosemary leaf extract’s antioxidant activity. Rosemary also contains other related diphenolic abietane diterpenes, rosmanol, epirosmanol, isorosmanol, rosmaridiphenol, and rosmariquinone, in very low quantities [49,50]. It is suggested that these related diterpenes may come from the oxidation of carnosic acid in the plants [51]. Due to the presence of an o-diphenol structure (two phenol groups in the *ortho*-position), carnosic acid is easily oxidised to *ortho*-quinone. Thus, carnosic acid is very unstable, leading to the enhanced formation of highly oxidized abietane diterpenes such as isorosmanol, dimethyl isorosmanol, and other related compounds, by enzymatic dehydrogenation and scavenging of activated oxygen [52].

The compound isolated from the band at *R*_F_ = 0.83 (compound **4**) has a similar ATR-FTIR spectrum to the spectrum of abietic acid (Figure 4d), especially to 15-hydroxy-7-oxodehydroabietic acid [53]. Abietane-type resin acids, such as abietic acid, are the major components of *Pinus* spp. wood resin [54]. Dehydroabietic acid is the major native resin acid present in the hexane extracts and is a chemical marker of *Pinaceae* family (*Pinus* spp.) [55]. However, although rosemary resembles *Pinus* spp. in both appearance and scent, it is a member of the mint family. The presence of diterpenic acids, such as 15-hydroxy-dehydroabietic acid in *Rosemary officinalis* L. has not been previously reported. The occurrence of diterpenic acids with high degree of oxidation such as 15-hydroxy-7-oxodehydroabietic acids (Figure 2c) can be explained by a degradation process corresponding to the ageing process. The C-H stretching absorption bands nearby 2900 cm^−1^ show a complex shape due to the presence of =CH, -CH_3_, -CH_2_ and -CH groups, where the -CH_2_ bands appear split due to the presence of conjugated C=C bonds [56]. Differences in the -OH structure also affect the 1700 cm^−1^ and 1200 cm^−1^ regions. The 1700 cm^−1^ band is broader due to the presence of hydrogen bonds between hydroxyls and carboxylic acid groups. Owing to the intermolecular bonds (seen in samples 2 and 3), the 1271 cm^−1^ band decreases while the 1239 cm^−1^ band rises (non-bonded and bonded C-O stretching).

## 3. Materials and Methods

### 3.1. Solvents and Chemicals

All reagents used were analytical grade. Ethyl acetate, 2,2-di(4-tert-octylphenyl)-1-picrylhydrazyl (DPPH·) free radical, gallic acid (97%), caffeic acid (98%), rosmarinic acid (≥98%), and abietic acid (≥75%), were obtained from Sigma-Aldrich (Castle Hill, Australia). Acetic acid, ethanol, methanol, were from Merck (Darmstadt, Germany), and anisaldehyde was from ACROS organics (Fair Lawn, NJ, USA). Milli-Q (Millipore) water was used to prepare all aqueous solutions.

### 3.2. Plant Extracts

Aerial parts of cultivated *Rosmarinus officinalis* L. (rosemary plant) were obtained from the local nursery. Dried leaves were ground to a fine powder and then extracted with ethyl acetate. Around 30 g of finely ground leaves were transferred into an Erlenmeyer flask and extracted five times by vigorous shaking for 15 min with 50 mL of solvent and filtered. The combined extracts were evaporated to dryness under vacuum. The mass (dried extract residue) was stored at −20 °C before use. A 10 mg/mL solution was prepared for HPTLC analysis using the extraction solvent that was used to prepare the extract.

### 3.3. Extractive Fermentation

A salting procedure using a 2.5% *w/v* sodium chloride solution as fermentation brine, was used to initiate fermentation with lactic acid bacteria (LAB) (or lacto-fermentation). Salt helps create an initial environment in which primarily salt-tolerant *Lactobacillus* thrives and produces enough lactic acid from sugars to prevent other bacterial cultures from growing. The initial inoculum of microorganisms for the fermentation was derived from the plant material’s microecosystem. Although LAB usually represents only a minor portion of the initial microecosystem, it tends to overtake the rest due to its metabolic capacity [57]. Approximately 30 g of ground fresh and 30 g of dried and ground rosemary plant material were soaked with a brine solution in separate Erlenmeyer flasks and left for two weeks to ferment. An acid fermentation started shortly after the herbal material was brined, evident by a few bubbles of generated carbon dioxide. After two weeks, the fermented material was extracted using 50 mL of ethyl acetate. After addition of the ethyl acetate, the mixture was mixed, allowed to stand for 1 h, and then placed in the freezer. The ethyl acetate was separated from the fermentation broth by fractional freezing of the mixture. After freezing, the water ice and plant component, precipitated into crystals while the ethyl acetate extract was in the liquid phase due to its much lower freezing point (−83 °C). The ethyl acetate extract was decanted and filtered. This whole process was repeated once again. The collected ethyl acetate filtrate was then evaporated to dryness. The yield of extract (extractable components), expressed on weight of plant material (leaves), was calculated from the following equation:

Yield (g/100 g) = (*W*_1_ × 100)/*W*_2_ (1)

where *W*_1_ is the weight of the dried extract residue obtained after solvent removal, and *W*_2_ is the weight of leaves taken for extraction. 10 mg/mL solutions of extract dissolved in ethyl acetate were then prepared for HPTLC analysis.

### 3.4. Lignin Extraction

Approximately 30 g of dried and ground rosemary leaves is treated with a 100 mL mixture of acetic acid/formic acid/water 30/55/15 (*v/v/v*), for 3.5 h at 105 °C. Under these conditions, lignin is dissolved, and hemicelluloses are hydrolyzed to oligosaccharides and monosaccharides. The concentrated extraction liquor obtained is then treated with water to precipitate the lignin present. Lignin is then removed by vacuum filtration through a sintered glass crucible (porosity grade 4), due to the minimal amount and fine precipitate of lignin residue in the filtrate.

### 3.5. Planar Chromatography

HPTLC separations were run on 20 cm × 10 cm normal phase Silica gel 60 F254 HPTLC glass plates (Merck, Darmstadt, Germany). Extracts (20 μL/band) were sprayed with compressed air as 8 mm bands onto the HPTLC plate by a Linomat 5 TLC sampler (CAMAG, Muttenz, Switzerland), with 14 mm distance from each side, and a minimum distance of 3 mm between tracks at 8 mm distance from the bottom. HPTLC separation was carried out with *n*-hexane-ethyl acetate-acetic acid (60:36:4, *v/v/v*) in an Automated Multiple Development chamber (AMD2, CAMAG, Muttenz, Switzerland) up to a migration distance of 80 mm, which took 20 min. Plates images were captured with a TLC Visualizer Documentation System (CAMAG, Muttenz, Switzerland) operated with winCATS software (CAMAG, Muttenz, Switzerland). Images were processed and evaluated using VideoScan Digital Image Evaluation software (CAMAG, Muttenz, Switzerland).

#### 3.5.1. Post Chromatographic Derivatization

Antioxidants were detected using the DPPH· assay reagent. The plate was sprayed with a 2.0 mg/mL solution of DPPH· in methanol. After incubation in the dark at room temperature for 30 min, antioxidants were visualized as bright zones against a purple background under white light. The antioxidant activity was expressed as µg of gallic acid equivalents (GAE) per 20 µL of extract by using the gallic acid calibration curve method [58].

Phenolic compounds were detected after derivatization with a 3% *w/v* neutralized ferric chloride solution in ethanol and heating the plate for 5 min at 110 °C. Neutralized ferric chloride solution is made by adding dilute NaOH solution, drop by drop, with shaking until a small but permanent brown precipitate of ferric hydroxide is obtained. The solution is filtered to remove precipitate and the clear filtrate is used for derivatization [59]. The content of total phenolics was expressed as µg of gallic acid equivalent per applied amount of extract per band (GAE µg/20 µL) [60].

Natural products, especially terpenes and steroids, were detected after derivatization with anisaldehyde/sulfuric acid reagent. The reagent was freshly prepared by mixing 0.5 mL of *p*-anisaldehyde with 10 mL glacial acetic acid, followed by 85 mL methanol and 5 mL concentrated sulphuric acid. After derivatization, the plate was heated for 10 min at 110 °C, or until maximum visualization of spots. The total amounts of steroids and terpenes present were expressed in sitosterol equivalents (SE) per 20 µL of extract by using a β-sitosterol calibration curve [60].

#### 3.5.2. ATR-FTIR Measurements

Attenuated total reflection-Fourier transform infrared (ATR-FTIR) spectra were measured on a Cary 630 FTIR spectrometer (Agilent Technologies, Santa Clara, CA, USA), interfaced with an ATR device with a diamond crystal plate (Pike Technologies, Madison, WI, USA). Spectra were recorded in the middle infrared (MIR) region, between 4000 and 600 cm^−1^ at 4 cm^−1^ nominal resolution and Happ-Genzel appodization, and 64 measurements were averaged. Obtained data were analysed using Resolution Pro FTIR spectroscopy software (version 5.2.0, Agilent Technology). A background spectrum of air was recorded under the same instrumental conditions before each sample reading.

The spectra of caffeic acid, carnosic acid, rosmarinic acid, abietic acid standards, together with extracted lignin, were recorded by placing a few drops of compound dissolved in a minimum amount of ethyl acetate on the diamond crystal. The sample spectrum was recorded after the solvent had completely evaporated.

For HPTLC-ATR spectra of compounds with significant antioxidant activity, approximately 100 mg of dried fermented extract was dissolved in 0.5 mL of ethyl acetate and applied as a single band onto an HPTLC plate. After plate development, 4 bands that had significant antioxidant activity in the DPPH· assay, with *R*_F_ values of 0.10, 0.37, 0.53, and 0.83, were scraped off the plate with a small spatula. The scrapings were washed with ethyl acetate, filtered to remove the silica, and the collected filtrate left overnight at room temperature to evaporate the solvent. The dried sample was dissolved in a few drops of ethyl acetate, placed on the ATR crystal, and the FTIR spectrum recorded after the ethyl acetate had evaporated.

## 4. Conclusions

Brining and lactic acid fermentation significantly enhanced antioxidant activity of rosemary leaves extracts by more than two-fold. This increased antioxidant activity was associated with increased extraction of phenolics, abietane diterpenoids and dehydroabietic acid. Furthermore, partial decomposition of lignin from solid parts of rosemary leaves during lacto-fermentation, releases low-molecular-weight aromatic compounds with antioxidant activity into the medium. Spontaneous lacto-fermentation of herbal medicines may enhance their biological activity by decomposition of complex substrates into compatible components that can be easily extracted, thereby improving its therapeutic potency. Thus, lacto-fermentation should be further investigated as a method of increasing the therapeutic potency of herbal medicines.

## Figures and Tables

**Table 1:** Antioxidant activity, total phenolics, and natural products contents in extracts.

	Assay	Antioxidant activity	Total phenolics	Natural products
		DPPH·	FeCl_3_	ASA
Extract		GAE (µg/band)	GAE (µg/band)	SE (µg/band)
UF		2.92	29.22	227.96
FFL		5.26	46.96	223.54
FDL		7.36	75.51	309.63

ASA = Anisaldehyde/sulfuric acid; UF = unfermented; FFL = fermented from fresh leaves; FDL = fermented from dried leaves.

**Table 2:** The effect of fermentation on the extraction of selected antioxidants and β-sitosterol.

	*R*_F_ = 0.10		*R*_F_ = 0.37		*R*_F_ = 0.76	
	Area	RAE	Area	CAE	Area	SE
	(pixels)	(µg/band)	(pixels)	(µg/band)	(pixels)	(µg/band)
UF	0	0	0	0	348279	64.39
FLF	14342	0.27	37313	1.84	245641	44.64
FDL	26184	0.98	55770	3.37	310522	57.13

RAE = rosmarinic acid equivalents; CAE = caffeic acid equivalents; UF = unfermented

**Table 3:** Calibration curves and methods validation.

Standard		Linear Regression Analysis	RSD	LOD (μg)	LOQ (μg)	Linear Range (µg/band)
Gallic Acid	DPPH·	*y* = 109028*x* – 20474 (*R*^2^ = 0.98)	3.98–8.48	0.33	1.12	0.4–5.0
Gallic acid	FeCl_3_	*y* = 10260*x* + 7849.4 (*R*^2^ = 0.99)	1.99–3.31	0.13	0.42	1.0–10
Sitosterol	ASA	*y* = 5195.2*x* + 13732 (*R*^2^ = 0.95)	2.6–6.87	0.43	1.48	0.5–8.0
Rosmarinic acid	FeCl_3_	*y* = 16637*x* + 9835.6 (*R*^2^ = 0.95)	3.51–5.95	0.21	0.73	0.5–7.0

## Data Availability

The data presented in this study are available on request from the corresponding author.

## Sample Availability

Samples of the rosemary extracts are available from the authors.
